# Human Infants' Preference for Left-to-Right Oriented Increasing Numerical Sequences

**DOI:** 10.1371/journal.pone.0096412

**Published:** 2014-05-06

**Authors:** Maria Dolores de Hevia, Luisa Girelli, Margaret Addabbo, Viola Macchi Cassia

**Affiliations:** 1 Université Paris Descartes, Laboratoire Psychologie de la Perception, CNRS, UMR 8158, Paris, France; 2 Cognitive NeuroImaging Unit, NeuroSpin, INSERM, U992, Saclay, France; 3 Universita' degli Studi di Milano-Bicocca, Department of Psychology, Milano, Italy; Langeveld Institute, Utrecht University, Netherlands

## Abstract

While associations between number and space, in the form of a spatially oriented numerical representation, have been extensively reported in human adults, the origins of this phenomenon are still poorly understood. The commonly accepted view is that this number-space association is a product of human invention, with accounts proposing that culture, symbolic knowledge, and mathematics education are at the roots of this phenomenon. Here we show that preverbal infants aged 7 months, who lack symbolic knowledge and mathematics education, show a preference for increasing magnitude displayed in a left-to-right spatial orientation. Infants habituated to left-to-right oriented increasing or decreasing numerical sequences showed an overall higher looking time to new left-to-right oriented increasing numerical sequences at test (Experiment 1). This pattern did not hold when infants were presented with the same ordinal numerical information displayed from right to left (Experiment 2). The different pattern of results was congruent with the presence of a malleable, context-dependent baseline preference for increasing, left-to-right oriented, numerosities (Experiment 3). These findings are suggestive of an early predisposition in humans to link numerical order with a left-to-right spatial orientation, which precedes the acquisition of symbolic abilities, mathematics education, and the acquisition of reading and writing skills.

## Introduction

The coding of ordinal information in spatial terms is a widespread phenomenon. For instance, most cultures picture the future as if it were ahead and the past behind, in reference to one's own body [1], [2]. One prominent spatial coding is that of numbers, which appears to be highly relevant for human innovation, as witnessed by the ubiquitous use of rulers, graphs, and other measurement tools. The idea that numbers are mentally represented in a stable spatial layout was first informally documented more than a century ago with the description of a series of ‘number forms’ or spatial configurations containing the series of numbers [3]. Nowadays, a growing amount of research on adults' numerical abilities is suggestive of the idea that numbers are represented as inherently variable distributions of activation over a spatially oriented number line [4]. Within this view, numbers correspond to different spatial extensions and positions along an oriented horizontal axis [5].

In its mature form, the association of numerical order and spatial orientation gives rise to a Spatial Numerical Association of Response Codes (or SNARC) effect, which refers to the advantage for a spatial congruency between numbers and responses [6]: Western adults respond faster to small numbers with the left hand, and to large numbers with the right hand (see also [7]). This phenomenon depends on the relative, rather than absolute, magnitude since the same number can be associated to opposite lateralized responses depending on the numerical interval in which it is embedded (e.g., ‘5’ is associated to the right in the ‘1–5’ interval, and to the left in the ‘5–9’ interval; [6]). Therefore, this spatially oriented mapping is not fixed but easily adapts depending on the task demands, being currently considered a strategy to mentally organize ordinal information [8], [9].

Numerous experimental paradigms have provided further evidence for a mapping whereby numbers are associated to different spatial positions, suggesting that the number-space mapping is not a mere association but it can impact our visuo-spatial processing. For instance, an Arabic digit prime in a detection task boosts attention orientation towards the left or right spatial hemifield depending on its magnitude [10]. Neuroimaging and neuropsychological studies also support the existence of this phenomenon. Partially overlapping regions in the parietal cortex are involved in both numerical and visuo-spatial tasks [11], [12], [13], and cortical areas associated to saccadic movements are recruited during arithmetical performance [14], suggesting that numerical processing drives participants' shifts of attention along a representational space [15]. Neuropsychological evidence shows that patients affected by spatial neglect show the same signature bias towards the right side of space for both visuo-spatial and numerical bisection tasks, i.e., identifying the centre of a physical line and the middle number in a numerical interval, respectively ([16], [17], [18]; but see [19], [20]). All in all, this body of research strongly supports the idea that, in human adults, numerical information and visuo-spatial processing share resources at both functional and neural levels [21]. Note, however, that the reported interaction between numbers and space is compatible both with the idea that numbers are converted to a spatial code by the brain, and with the idea that numbers are associated to a spatial code, but are themselves distinct.

The prevalent view on the origins of this oriented number-space mapping has been that culture determines the association between numerical magnitudes and spatial positions along the representational continuum. This view was supported by the finding that adults with opposite reading/writing directions, such as Western vs. Arabic, exhibit an opposite SNARC effect, left-to-right vs. right-to-left oriented, respectively [6], [22], [23]. Moreover, the SNARC effect was originally described only in older children, i.e., 9-year-olds, who had already been well introduced to the reading system and to mathematical education [24], supporting the view that the number-space mapping is a product of enculturation.

While we are gaining a rich insight into the developmental origins of numerical abilities, which trace back to the very beginning of postnatal life [25] and are relatively well understood in infancy [26], [27], [28], [29], [30] and childhood [31], [32], [33], [34], our knowledge regarding the origins of the spatialization of numbers is currently very limited. Recently, however, developmental research has provided new insights into this question. First, it has been shown that the spontaneous mapping between number and space is present in the preschool years ([35]; for a discussion, see [36], [37]), with 4- to 5-year-old children showing the same signature bias towards the larger number, as adults do, in a line bisection task with numerical flankers [38],[39],[40]. Second, and more critical, preschool children exhibit intuitions of an oriented numerical continuum: Western 3- to 4-year-old children expect numerical transformations to be congruent with a left-small and right-large number-space association [41], they start to count from the left [42], and judge small non-symbolic numbers faster when presented on the left side of space and large non-symbolic numbers on the right side of space [43]. These findings show that, although cultural factors may play a role before the start of formal schooling, well-established reading/writing abilities are not essential for the mental association between numbers and an oriented spatial representation. This is also supported by evidence from a recent provocative study conducted with non-human animals showing that a spontaneous tendency to associate large number to the right side of space is present in newly-hatched chicks, who manifested a rightward bias in an arithmetic task in which they were required to search for the larger number of objects in a pair [44].

More critical to the origins of the number-space mapping would be evidence from the infant population, for whom the effects of enculturation are minimized and who lacks symbolic tools. Recent attempts to reveal a number-space mapping in infancy have led to promising findings. Eight-month-old infants transfer the discrimination of an ordered series of non-symbolic numbers to an ordered series of line lengths, and successfully learn and generalize a rule that establishes a positive relationship between non-symbolic number and length, while they fail with an inverse relationship [45]. The transfer of discrimination from number to spatial extent is bidirectional and extends to the dimension of temporal duration in 9-month-old infants [46]. Recently, the mapping of number, space and time has been found to be present even at birth [47]. Overall, these findings show that, just like adults do, preverbal infants map number onto a corresponding spatial extent (and the reverse).

However, whether in infants these spatial extents are linked to different positions along a horizontal continuum, like in adults, is still an open question. Suggestive examples of an unlearned mapping between number and horizontal spatial positions come from studies on non-human populations. When newly-hatched chicks are trained to peck at the 4^th^ position of a vertically-oriented series of tokens, they later show a spontaneous, significant leftward bias once the tokens have been horizontally arranged [48]. This leftward attentional bias holds whenever spatial and numerical information are congruent across conditions [49], and it has been attributed to right hemispheric dominance in visuo-spatial processing, which is thought to favor allocation of attention to the left hemifield. Animal models provided by fish further support this view showing that, by experimentally manipulating the direction of hemispheric lateralization, it is possible to modulate the direction of spatial biases observable while animals perform a bisection task [50]. Overall, these findings from non-human, non-linguistic species highlight the role of neural factors and visuo-spatial processing strategies in engendering attentional biases. These automatic biases might be at the origins of an oriented mental number line.

In fact, in human infants there is evidence for a timing asynchrony in the functional maturation of cerebral hemispheres, with the right hemisphere developing faster than the left one during prenatal and postnatal life (for a review, see [51], [52]). As proposed for fish and chicks, these spatio-temporal constraints on brain development may determine an asymmetrical exploration of visual space in the earlier stages of human postnatal life. Furthermore, it is possible that such a leftward bias might constrain not only the exploration of external space, but also the structure of infant's representational space, in which stored information would be organized along a spatial continuum originating from the left side. In fact, it is known since the seminal studies by Haith [53] that at birth, and during very early months of life, horizontal scans are wider and more frequent than vertical ones, with visual exploration and stimulus detection being easier along the horizontal than the vertical axis and looking times in preferential looking tasks being longer to horizontal than to vertically oriented stimuli [54], [55]. An automatic exploration of external and internal space from left to right might be also present in infancy and contributes to the emergence during development of an oriented mental number line.

Presence of ordinal abilities in infancy might be a prerequisite for a relationship between numerical order and spatial orientation to emerge. The ability to order information in sequences is, in fact, critical to trigger the use of an oriented spatial representation in adults, for whom different examples have been reported: Tone height [56], letters of the alphabet [57], temporal events [58], and unrelated words that are memorized in a specific order [59], all are internally represented along an oriented space, as reflected by the observation of SNARC-like effects in the processing of these dimensions. This evidence suggests that a spontaneous tendency to associate numbers to oriented spatial codes might be present whenever infants manifest the ability to process and represent ordinal information.

Various lines of evidence suggest that this ability is available from the very beginning of postnatal life [60], [61], and that by 8–11 months of age infants are able to process complex spatiotemporal sequences of objects moving along different locations [62]. By the second half of the first year of life, infants detect and discriminate ordinal relations among magnitudes for dimensions such as number and size [63], [64], [65], [66]. Critically, at the youngest age at which this ability has been reported, i.e., 4 months, infants discriminate increasing but not decreasing size-based sequences [64]. This developmental advantage for increasing vs. decreasing order resembles ordinal learning in nonhuman primates [67], [68], and might be a precursor of the advantage for addition over subtraction in non-symbolic and symbolic arithmetic in children and adults [69], [70], [71], [72]. By 7 months of age, this asymmetry is no longer present, with infants successfully discriminating both increasing and decreasing numerical sequences [65].

Overall, research with preverbal infants provides evidence for an early representational mapping between non-symbolic number and spatial extent [45], [46], an early sensitivity to ordinal information [60], [61], [65], and an early advantage for increasing magnitude [64], [73]. Capitalizing on this evidence, in the current study we investigated whether at 7 months of age numerical order is spontaneously related to a left-to-right oriented spatial representation, as in non-human animals [48] and human adults [6]. We tested infants' ability to detect and represent increasing and/or decreasing numerical order within spatiotemporal sequences (i.e., left-to-right or right-to-left oriented). Previous research has shown that 7-month-old infants discriminate the ordinal relations (increasing vs. decreasing) embedded in a series of three, sequentially-presented numerical displays in the absence of spatial cues [65]. In the present study we tested whether infants maintain their ordinal abilities when directional spatial cues are added to the numerical sequences. More crucially, we investigated whether the impact of these directional spatial cues on ordinal abilities varies as a function of the specific orientation along which they are provided (i.e., left-to-right vs. right-to-left). We habituated infants to increasing or decreasing numerical displays embedded in spatiotemporal sequences oriented either from left to right (Experiment 1), or from right to left (Experiment 2), and tested infants' ability to discriminate an inversion in ordinal direction. We hypothesized that if the two orientations along which numerical information is presented (i.e., left-to-right vs. right-to-left) have differential impact on infants' processing of numerical order, with infants favoring a left-to-right oriented numerical series over a right-to-left oriented one, just like newborn chickens [48] and human adults [6] do, then infants should perform differently in Experiment 1 vs. Experiment 2.

We tested 7-month-old infants using the same methods that revealed successful discrimination of numerical order in infants of the same age in the past [65]. The only difference between the current and past experiments is that we presented the ordered numerical displays at different spatial locations along either a left-to-right oriented (Experiment 1) or a right-to-left oriented (Experiment 2) horizontal axis.

## Experiment 1

In Experiment 1 infants were habituated to either increasing or decreasing numerical sequences, and were tested with new sequences displaying both the familiar and the novel orders. During both the habituation and test trials, the first numerical array appeared on the left, the second on the centre, and the third one on the right side of the screen, so that increasing/decreasing numberwas explicitly associated to a horizontal spatial displacement oriented from left to right.

### Methods

#### Participants

Participants were 24 healthy, full term 7-month-old infants (mean age = 7 months, 18 days; range = 7 months, 0 days – 8 months, 0 days; 16 female). Nine additional infants were tested but excluded from the final sample because of fussiness (N = 6) or being uncooperative (N = 3) resulting in failure to complete all test trials. Infants were recruited via a written invitation sent to parents based on birth records provided by the neighboring cities. The majority of participants were from Caucasian, middle class families.

#### Ethics Statement

The experiment was conducted after obtaining Institutional Review Board approval from the Department of Psychology at the University of Milano-Bicocca. All participants' parents gave informed written consent before testing began.

### Stimuli

Stimuli were arrays of colored rectangular-shaped items presented on a white background, randomly arranged, with the item's shorter side aligned with the horizontal plane. There were four sets of stimuli, three for the habituation phase and one for the test phase. The three habituation sets were composed of numerical arrays containing 6, 12, and 24 items; 9, 18, and 36 items; and 12, 24, and 48 items, respectively. The numerical arrays composing the test stimulus set contained 4, 8, and 16 items. Each stimulus set was presented in a different color: “blue” (rgb: 0, 0, 255) for the 6-12-24 set, “red” (rgb: 255, 0, 0) for the 9-18-36 set, “green” (rgb: 6, 141, 6) for the 12-24-48 set, and “purple” (rgb: 201, 28, 195) for the 4-8-16 test set. For each stimulus set, three different exemplars that differed in item configuration were generated.

**Figure 1:**
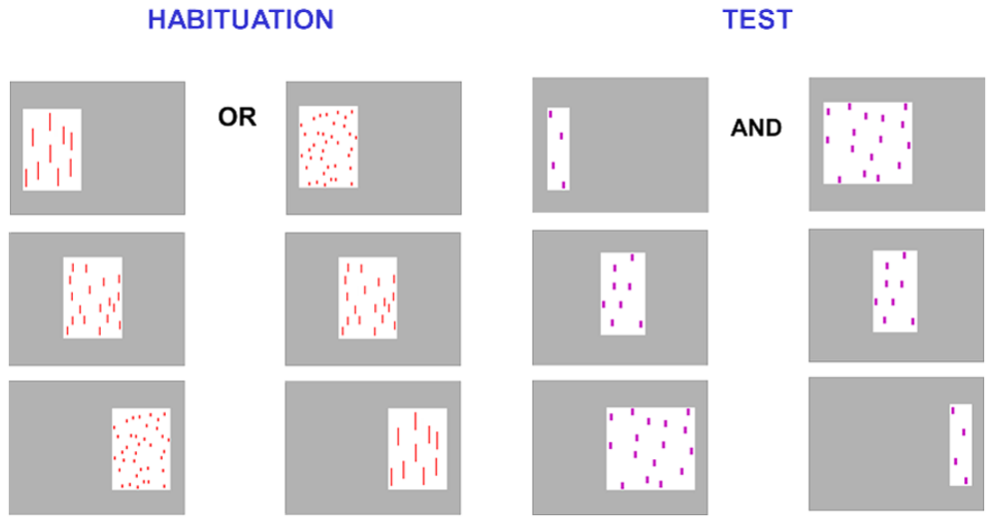
Examples of stimuli used in Experiment 1. During habituation infants are presented with either increasing or decreasing numerical sequences (e.g., 9-18-36 or 36-18-9), and at test all infants are shown both increasing and decreasing new numerical sequences (e.g., 4-8-16 and 16-8-4). All sequences in both habituation and test are presented from left to right.

Non-numerical, continuous cues were controlled for, with cumulative surface area and contour length being constant during habituation by varying item size and shape inversely to number. Thus, the heights of the single items for the small, medium, and large displays were, respectively, 3.3 cm, 1.5 cm, and 0.6 cm, with the width constant at 0.3 cm. The size of each habituation display was held constant at 176.04 cm^2^ (16.3 cm × 10.8 cm), so that number covaried with density. For test displays, the cumulative surface area and contour length were positively correlated with number, so that item size was kept constant across numbers (1.4 cm × 0.5 cm), and the display size varied, with density held constant at 0.06 elements per cm^2^ (Figure 1). In this way, the continuous, non-numerical variables that varied during habituation were held constant during test, and vice-versa.

### Design and Procedure

Half of the infants, randomly assigned, were habituated to the increasing number sequences, the other half to the decreasing number sequences. Within each habituation condition, the three different stimulus sets were cycled in a fixed order until the infant met the habituation criterion: from the smallest to the largest numerical display for the increasing condition (i.e., 6-12-24; 9-18-36; 12-24-48), and from largest to the smallest for the decreasing condition (i.e., 48-24-12; 36-18-9; 24-12-6). Following habituation, all infants viewed six test trials with new numerical sequences alternating increasing and decreasing sequences. Test order was counterbalanced across participants.

Infants were installed in an infant seat at approximately 60 cm from the monitor where the stimuli were presented. A curtain separated the participant from the experimenter at all times. Parents remained on the experimenter's side of the curtain, so as to not distract the infant, but were free to approach the infant at any time should he or she show signs of distress. A video camera, directed to the infant's face, was positioned just above the stimulus presentation monitor. The live image of the infant's face was displayed on a TV monitor to allow the online coding of the infant's looking times by the experimenter, who was blind to the habituation condition to which the infant was assigned. The live image of the infant's face was recorded, so that for half of the infants in each habituation condition, randomly selected, data were subsequently coded offline. Inter-coder agreement (Pearson correlation) between the two observers who coded the data live or from digital recording as computed on total fixation times on each of the six test trials was *r* = .983.

Before starting each trial, a cartoon-animated image associated to a varying sound appeared on the screen. As soon as the infant looked in the direction of the animated image, the experimenter started the trial. Each trial consisted in a repeating cycle (5750 ms in total) composed of a black screen (500 ms) followed by the three numerical displays. The displays were consecutively presented on a white background for 1750 ms each. Each trial continued until the infant looked for a minimum of 500 ms and ended when the infant looked away continuously for 2 s or looked for a maximum of 120 s. The three habituation sequences were presented in a fixed order and repeated until the infant either was given a maximum of 14 trials or met the habituation criterion, which was defined as a 50% decline in looking time on three consecutive trials, relative to the total looking time on the first three trials that summed to at least 12 s. Following habituation, infants were given six test trials, in which novel and familiar ordinal sequences appeared in alternation, with half of the infants seeing the novel test sequence first.

The three numerical displays were consecutively presented (for 1750 ms each) on a grey background in three different spatial positions. The first display appeared always on the left side of the screen, (which corresponded to the area spanning 7 cm from the left edge of the screen and 36.5 cm from the right edge of the screen), the second display appeared within the central area of the screen (22 cm halfway between the edges), and the third display appeared on the right side of the screen (36.5 cm from the left edge of the screen and 7 cm from the right edge of the screen).

In order to familiarize infants with the spatial task and calibrate the infants' gaze, they saw a cartoon-animated image associated to a sound that appeared sequentially on the left and on the right positions of the screen. The animated image appeared first on the left side of the screen, and, as soon as the infant looked in that direction, the experimenter presented the same image on the right side of the screen. This calibration served both for the coding of the infant's looking behaviour and to equally direct infant's attention to both sides of the screen.

### Results

All infants habituated to the decreasing numerical sequences and 10 out of 12 infants habituated to the increasing numerical sequences reached the habituation criterion. An ANOVA with habituation condition (increasing vs. decreasing) as the between-subjects factor, and habituation trials (first three vs. last three) as the within-subjects factor revealed a significant effect of habituation trials, *F*(1,22) = 75.99, *p*<.0001, due to average looking time on the first three habituation trials (*M* = 21.82 s) being significantly longer than average looking time on the last three habituation trials (*M* = 7.84 s). There was no main effect or interaction involving habituation condition (both *F*s<1, n.s.). No differences in overall looking time and number of trials to habituate were found across the two habituation conditions: For the increasing numerical sequences, infants required an average of 128.9 sec. and 8.6 trials to habituate, for the decreasing numerical sequences, infants required 111.9 sec. and 7 trials (unpaired t-tests, both *t*s<1.8, n.s.).

**Figure 2:**
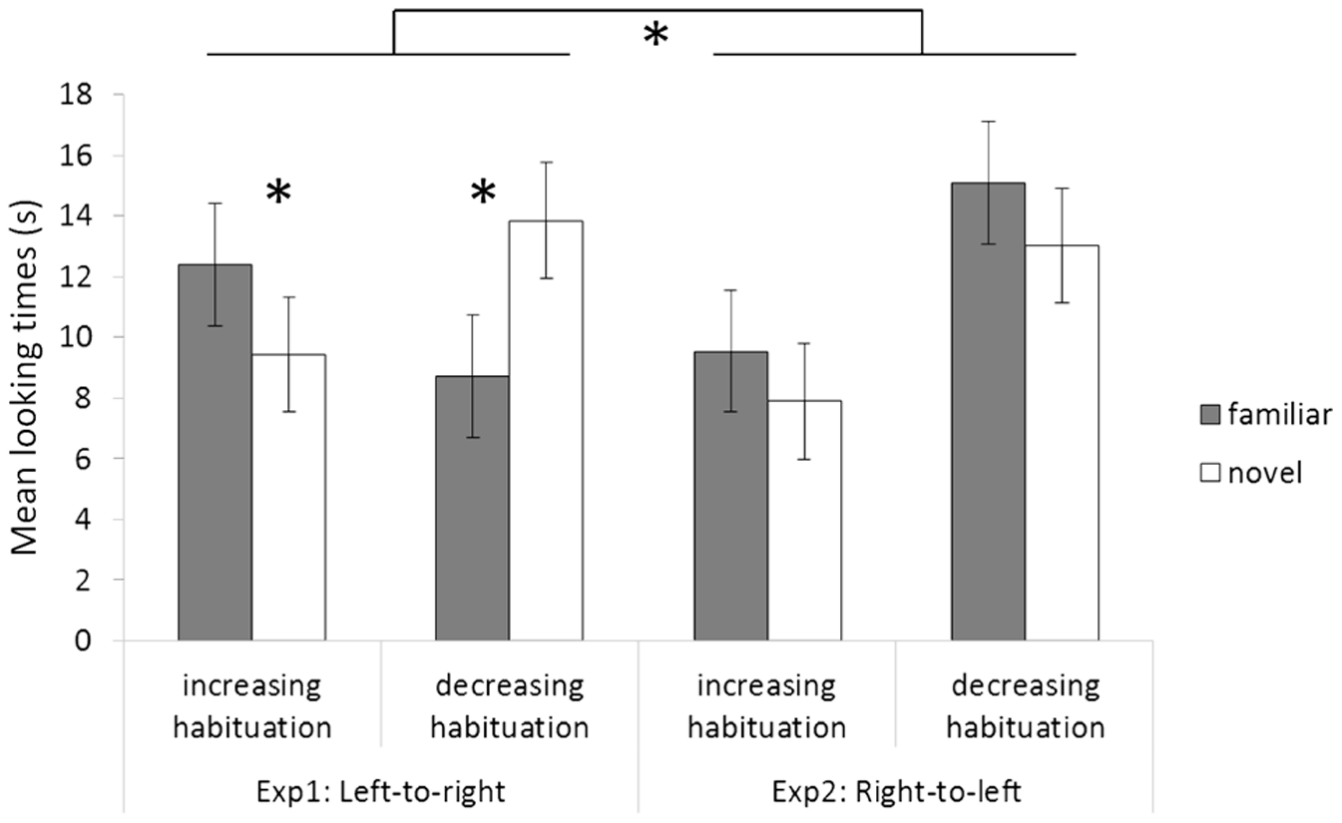
Looking times in Experiment 1 and Experiment 2. Mean looking times and s.e. (seconds) to the familiar and to the novel test trials for infants habituated to increasing and for infants habituated to decreasing sequences for both Experiment 1 and Experiment 2.

An ANOVA with habituation condition (increasing vs. decreasing) and first test trial (familiar vs. novel) as between-subjects factors, and trial pair (first vs. second vs. third) and test trial type (familiar vs. novel ordinal direction) as within-subjects factors was performed on total looking times during test trials. There was a significant main effect of trial pair, *F*(2,40) = 8.83, *p*<.001, *η_p_^2^* = .306; infants looked longer to the first (*M* = 14.04 s, *SD* = 5.8) relative to the second (*M* = 9.94 s, *SD* = 6.0, *p*  = .001) and to the third pair of trials (*M* = 9.3 s, *SD* = 4.9, *p*<.001), indicating an order effect in which a progressive decrease in looking times arises with time. A significant Test trial type x Habituation condition interaction, *F*(1,20) = 18.01, *p*<.001, *η_p_^2^* = .474, revealed through LSD post-hoc comparisons that infants in the increasing habituation condition looked longer to the familiar than to the novel trials at test (*M* = 12.4 s, *SD* = 4.9 vs. *M* = 9.4 s, *SD* = 5.7; *p*<.05), whereas infants in the decreasing habituation condition looked longer to the novel than to the familiar trials at test (*M* = 13.8 s, *SD* = 5.3 vs. *M* = 8.7 s, *SD* = 4.3; *p*  = .001) (Figure 2). Eleven out of 12 infants (*p*<.01, binomial test) in the increasing habituation looked longer to the familiar test trial, and 10 out of 12 (*p*<.05, binomial test) infants in the decreasing habituation condition looked longer to the novel test trial. Therefore, infants in both habituation conditions were able to discriminate the novel from the familiar ordered sequences, as shown by their different looking times during posthabituation test trials. Critically, the direction of posthabituation preference was reversed in the two habituation conditions: infants habituated to decreasing sequences preferred the novel (increasing) order, whereas infants habituated to increasing sequences preferred the familiar (increasing) order. Therefore, all infants showed significantly longer looking times to the increasing left-to-right oriented sequence, irrespective of the habituation condition to which they were previously exposed.

### Discussion

The results of Experiment 1 show that, irrespective of the direction of numerical order to which they had been habituated, infants discriminated between the familiar and novel orders at test when numerical displays were spatially displaced along a left-to-right orientation. Nevertheless, infants performed differently from previous studies where the same stimuli had no spatial information [65]. Although all infants detected the inversion in ordinal direction between the familiar and novel sequences at test, their looking behavior varied depending on their habituation: When habituated to increasing order, infants looked significantly longer to the familiar order at test, while when habituated to decreasing order, infants looked significantly longer to the novel order at test.

Although infants typically exhibit robust preference for novelty relative to familiarity within habituation-dishabituation, they can also exhibit familiarity preferences under several circumstances, such as when the stimuli are complex rather than simple (e.g., [74]), when infants had insufficient habituation time to form a strong representation of the stimulus, or when a pre-experimental preference for the familiar over the novel stimulus exists, which interferes with the experimentally driven novelty preference (e.g.,[45], [54], [75]). Because almost all infants (10 out of 12) in the increasing habituation condition reached the habituation criterion, and required similar amounts of looking time and number of trials in order to habituate, compared to infants in the decreasing habituation condition, it is unlikely that incomplete habituation may explain the familiarity preference at test. Rather, results of Experiment 1 are compatible with the presence of a spontaneous preference for increasing spatiotemporal sequences in which numbers appear from left to right. Since no preference for increasing over decreasing order in the absence of spatial information has been reported before with similar methods [65], results suggest that spatial displacement may have enhanced the saliency of increasing order with respect to decreasing order in the current experiment (see [64], [73]). Before testing for a baseline preference for left-to-right increasing sequences in Experiment 3, we asked in Experiment 2 whether a right-to-left spatial orientation would have the same impact on infants' ordinal abilities as found in Experiment 1. In light of existing evidence showing cultural modulation of the direction of the number-space mapping in children [76] and adults [64], [73], it is possible that numerical order in preverbal infants might be equally associated to left-to-right and to right-to-left orientations.

## Experiment 2

In Experiment 2 infants were habituated to either increasing or decreasing right-to-left oriented numerical sequences, and were tested with new numerical series in both familiar and novel orders. In both the habituation and test trials the first numerical array of each sequence appeared on the right, the second on the centre, and the third one on the left side of the screen, so that increasing/decreasing number was associated to a horizontal spatial displacement from right to left.

### Methods

Stimuli design, apparatus, and procedure were the same as in Experiment 1, except for the following.

#### Participants

Participants were 24 healthy, full term 7-month-old infants (mean age = 7 months, 16 days; range = 7 months, 3 days – 7 months, 29 days; 14 female). Data from 7 additional infants were discarded because of looking times in at least one test trial shorter than 1 s (N = 2), fussiness (N  = 3) or being not cooperative (N = 2), resulting in failure to complete all the test trials.

### Procedure

**Figure 3:**
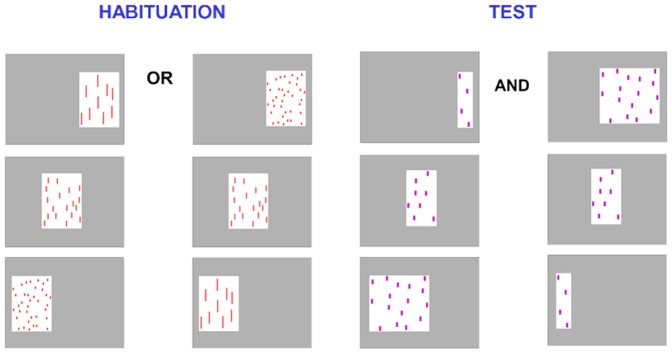
Examples of stimuli used in Experiment 2. During habituation infants are presented with either increasing or decreasing numerical sequences (e.g., 9-18-36 or 36-18-9), and at test all infants are shown both increasing and decreasing new numerical sequences (e.g., 4-8-16 and 16-8-4). All sequences in both habituation and test are presented from right to left.

The only difference with Experiment 1 was that the first display appeared always on the right (7 cm from the right edge of the screen and 36.5 cm from the left edge of the screen), the second on the centre (22 cm halfway between the edges), and the third on the left side of the screen (36.5 cm from the right edge of the screen and 7 cm from the left edge of the screen) (Figure 3).

The animated image used during the familiarization/calibration phase appeared first on the right side of the screen and, as soon as the infant looked in that direction, the experimenter presented the same image on the left side of the screen. Inter-coder agreement (Pearson correlation) was *r* = .993.

### Results

Ten out of 12 infants in the increasing habituation condition, and also 10 out of 12 in the decreasing habituation condition, reached the habituation criterion. An ANOVA with habituation condition (increasing vs. decreasing) as the between-subjects factor, and habituation trials (first three vs. last three) as the within-subjects factor revealed a significant main effect of habituation trials, *F*(1,22) = 29.93, *p*<.0001, due to average looking time during the first three habituation trials (*M* = 15.3 s) being significantly longer than average looking time during the last three habituation trials (*M* = 7.6 s). There was no main effect or interaction involving the factor habituation condition (both *F*s<1, n.s.). Overall, infants in the increasing habituation condition required an average of 84.4 s and 7.7 trials to reach the habituation criterion, those in the decreasing habituation condition required 93.4 s and 8.3 trials (unpaired t-tests, both *t*s<1, n.s.).

Total looking times during test trials were analyzed by means of a four-way ANOVA with habituation condition (increasing vs. decreasing) and first test trial (familiar vs. novel) as between-subjects factors, and trial pair (first vs. second vs. third) and test trial type (familiar vs. novel ordinal direction) as within-subjects factors. The ANOVA revealed a significant interaction between trial pair and first test trial, *F*(2,40) = 4.47, *p*<.05, *η_p_^2^* = .183. This interaction was due to looking times being longer during the second pair of test trials (*M* = 17.7 s, *SD* = 15.7), relative to the first (*M* = 9.9 s, *SD* = 7.4; *p*<.01) and to the third ones (*M* = 11.9 s, *SD* = 6.7; *p*<.05) for infants starting with the novel test. No main effect of the factor test trial type, *F*(1,20) = 1.6, *p* = .2, *η_p_^2^* = .076, or interactions involving this factor (all *F*s<3.2, *p*s>.09), approached statistical significance (see Figure 2). Eight out of 12 infants (*p* = .38, binomial test) in the increasing habituation condition looked longer to the familiar test trial, and 5 out of 12 infants (*p* = .77, binomial test) in the decreasing habituation condition looked longer to the novel test trial. Therefore, infants presented with right-to-left oriented numerical sequences were not able to discriminate between the two orders at test, suggesting that spatial information hindered infants' ordinal abilities in the present experiment.

In order to directly compare the performance from Experiments 1 and 2, we ran an ANOVA with spatial orientation (left-to-right vs. right-to-left) and habituation condition (increasing vs. decreasing) as between-subjects factors, and test trial type (familiar vs. novel) as within-subjects factor. The analysis revealed the presence of a significant Habituation condition x Test trial type interaction, *F*(1,44) = 4.74, *p*<.05, *η_p_^2^* = .108, as well as a significant triple interaction between spatial orientation, habituation condition and trial test type interaction, *F*(1,44) = 5.81, *p* = .02, *η_p_^2^* = .130, confirming that infants showed different looking behavior during the test trials across the two experimental conditions (see Figure 2). In contrast, unpaired t tests on total looking times and number of trials to habituate showed that infants spent a similar amount of time and received a similar number of trials across the two experiments (all *p*s>.08).

### Discussion

Infants in Experiment 2 did not show evidence of being able to extract ordinal information from right-to-left oriented numerical sequences. Because infants have been found to manifest the ability to discriminate ordinal numerical sequences in the absence of spatial cues [65], these findings suggest that it is the spatial dislocations of numerical arrays along a right-to-left oriented horizontal axis that disrupted infants' sensitivity to ordinal information in the current experiment.

Crucially, results of Experiment 2 contrast with those of Experiment 1 where, with a left-to-right oriented presentation of the ordered sequences, infants were able to discriminate at test between the familiar and the novel order. Differences in infants' performance in Experiment 1 vs. Experiment 2 cannot be accounted for by the numerical information presented, since the same numerical arrays were used in both experiments, nor by different exposure to the ordinal information during the habituation phase, since there was no difference in looking time and number of trials to habituate between the two experiments. Therefore, the only variable that can account for the contrasting performance in the two experiments is the specific spatial orientation along which the numerical displays were presented, either from left to right (Experiment 1) or from right to left (Experiment 2).

When considered together, results of Experiments 1 and 2 point to a specific coupling between numerical order and spatial orientation, which may drive a baseline preference for increasing over decreasing numerical sequences when numerical information is provided along a left-to-right orientation. In fact, as discussed above, infants' preference for the familiar test trials in Experiment 1 might imply an a priori preference for left-to-right oriented increasing numerical sequences. We explored this possibility in Experiment 3 by using a visual preference method (see [45], [54]), in which infants were presented with both increasing and decreasing numbers along a left-to-right orientation without previous habituation to any numerical order. This experiment allowed us to test the presence of a spontaneous preference for increasing numerical sequences that are spatially arranged from left to right.

## Experiment 3

In Experiment 3 infants were shown two blocks of trials displaying the same increasing and decreasing numerical sequences used in the habituation phase of Experiments 1 and 2, with increasing and decreasing order alternated between blocks. All sequences were presented in a left-to-right spatial orientation, without previous habituation to any sequence.

### Methods

Methods and procedure were the same as in Experiment 1, except as follows.

#### Participants

Participants were 20 healthy, full term 7-month-old infants (mean age  = 7 months, 17 days; range  = 7 months, 2 days – 7 months, 28 days). Eight of the participants were female. Thirteen additional infants were tested but excluded from the final sample due to looking times in at least one test trial shorter than 1 s (N = 2), fussiness (N = 5) or being not cooperative (N = 3), resulting in failure to complete all the test trials. Data from 3 additional subjects were excluded due to excessively long looking times on one test trial (greater than 3 SD away from the mean looking time of all infants).

### Stimuli and Procedure

**Figure 4:**
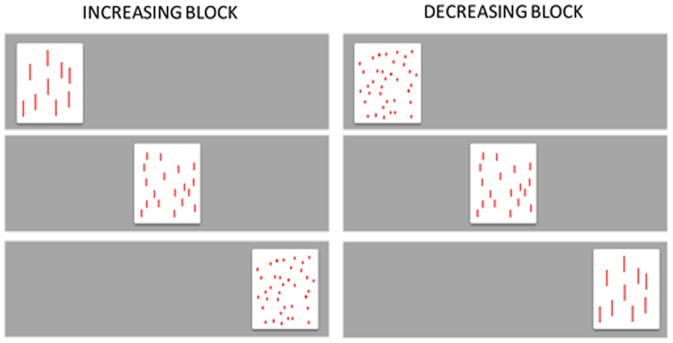
Examples of stimuli used in Experiment 3. Infants are presented with two blocks of trials presenting either increasing or decreasing numerical sequences, all left-to-right oriented.

The numerical displays were the same used in the habituation phase of Experiments 1 and 2. Infants received six trials grouped in two blocks: one block of three trials displaying increasing sequences and the other three-trial block displaying decreasing sequences. All sequences were left-to-right oriented, and order of blocks was counterbalanced across participants (Figure 4). Looking time to each trial was recorded in the same way as in previous experiments. Inter-coder agreement (Pearson correlation) was *r* = .967.

### Results and Discussion

**Figure 5:**
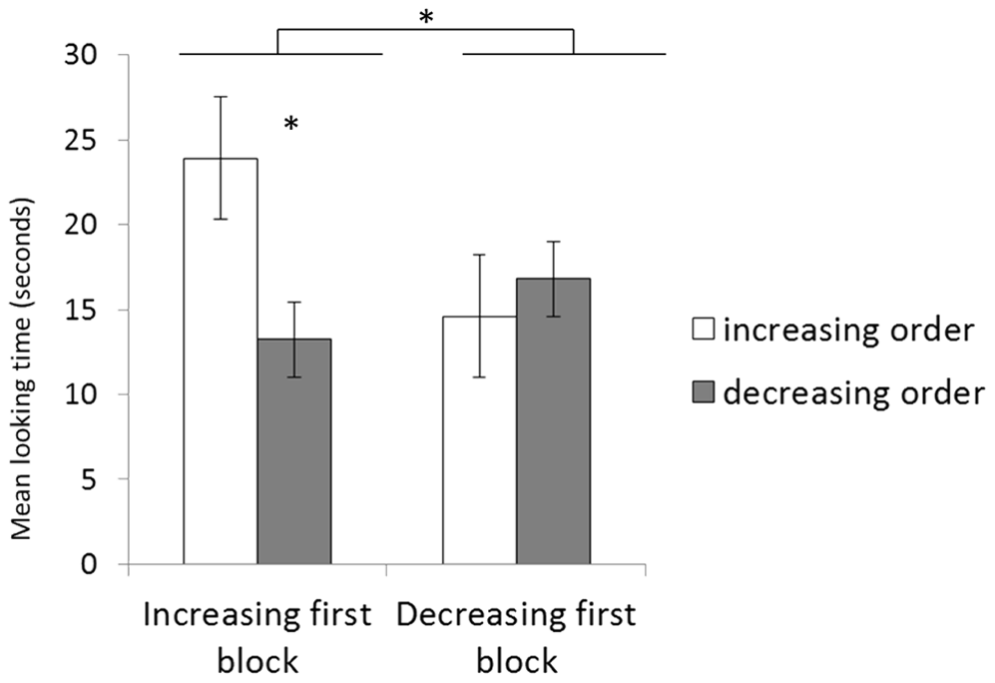
Looking times in Experiment 3. Mean looking times and s.e. (seconds) to increasing and decreasing numerical sequences presented along a left-to-right orientation, for infants presented with the increasing block first and for infants presented with the decreasing block first.

For each participant, mean looking time for each block of trials was calculated and entered into an ANOVA with first block (increasing first vs. second) as the between-subjects variable and ordinal direction (increasing vs. decreasing order) as the within-subjects variable. There was a significant main effect of ordinal direction, *F*(1,18) = 4.44, *p*<.05, *η_p_^2^* = .198; infants looked longer to the increasing (*M* = 19.2 s, *SD* = 2.5) than the decreasing trials (*M* = 15 s, *SD* = 1.6). The interaction between ordinal direction and first block was also significant, *F*(1,18) = 10.37, *p*<.005, *η_p_^2^* = .366. When explored through LSD post-hoc comparisons, this interaction revealed that infants looked longer to the trials showing the increasing left-to-right oriented sequences when they received these trials in the first block (*p*<.01), but not in the second one (*p* = .4). Critically, this finding was not due to higher looking to the first block irrespective of the ordinal information, since infants receiving the decreasing block first did not show any preference (Figure 5). Looking times to the decreasing order in these infants might have been washed off by the preference for the increasing block or dispreference for the decreasing block. Examination of the data for individual infants confirmed the results on average looking times, showing that 9 out of the 10 infants who received the increasing block first looked longer to the increasing sequences (*p*<.01, binomial test), whereas only 6 out of the 10 infants who received the decreasing block first looked longer to the decreasing sequences (*p*>.2, binomial test).

Overall, results from Experiment 3 are suggestive of a spontaneous preference for numerical increasing sequences spatially oriented from left to right over decreasing sequences with a similar left-to-right orientation, as also suggested by infants' performance in Experiment 1. However, this preference appears to be malleable, since infants showed a modulation of looking times as a function of which block they received first, with infants who were first exposed to decreasing left-to-right oriented sequences failing to show a preference for the increasing ones. This finding is in line with the idea that the early bias to link numerical order to spatial directionality is plastic and easily modifiable by experiential and cultural factors, as suggested by observations that the specific orientation of the number-space mapping in children [22], [76] and adults is dependent upon exposure to conventional routines, mostly dependent on the dominant reading/writing system [22].

## General Discussion

The present study investigated the presence in infancy of an association between numerical order and spatial left-to-right orientation. At 7 months, infants are able to extract ordinal information from numerical sequences when no spatial information is provided [65]. In the current study we investigated 7-month-olds' ordinal numerical abilities in the presence of spatial information, by presenting spatiotemporal numerical sequences in which numerical arrays appeared at different spatial locations along an oriented horizontal axis, either from left to right (Experiment 1) or from right to left (Experiment 2).

Overall, our findings indicate that discrimination of ordinal numerical sequences is impacted when spatial information is embedded within the sequences. In Experiment 1, infants were shown ordinal numerical sequences appearing along a left-to-right oriented axis, so that numerical ordering was explicitly associated both to an increasing and a decreasing left-to-right orientation. In Experiment 2, infants were shown the exact same numerical stimuli as in Experiment 1 but displayed along a right-to-left spatial orientation. While infants in Experiment 1 showed significant differences in looking times between increasing and decreasing order during the test trials, infants in Experiment 2 showed no indication of discrimination during post-habituation trials, suggesting that spatial direction has a critical impact on infants' ability to extract ordinal information from numerical sequences. The absence of a mapping of decreasing numerical sequences onto right-to-left space in Experiment 2 indicates that infants at 7 months do not relate numerical magnitude to lateralized spatial codes (i.e., small/left and large/right) as adults do, and therefore our findings do not support the existence of an oriented number-space mapping in infancy. Future research should determine whether the inability to track and represent objects presented from right-to-left is specific to numerical information or whether it extends to other types of information.

A crucial finding from Experiment 1 is that all infants preferred increasing left-to-right sequences to decreasing sequences at test, irrespective of the ordinal direction to which they were habituated, suggesting the presence of a baseline preference for increasing numerical order oriented in space from left to right. This possibility was tested in Experiment 3 using a visual preference procedure, in which infants were presented with alternating blocks of trials showing increasing and decreasing left-to-right oriented sequences, in the absence of a habituation phase. In this task, infants who were first presented with increasing numerical sequences showed a preference for these sequences, whereas infants who first received decreasing sequences did not manifest any preference. This asymmetry in infants' responses suggests that the bias toward left-to-right oriented increasing order is not very robust at 7 months, and therefore easily malleable. This would leave a lot of space for cultural and experiential factors to either strengthen the bias or override it, eventually giving rise to culturally dependent strategies to represent numerical order.

Overall, the present findings provide evidence that 7-month-old infants relate numerical order and spatial orientation. This evidence sheds light on the developmental origins of the oriented number-space mapping observed in adults, suggesting that the spatialization of numerical information is built on an early preference for increasing numerical order presented from left to right. This early preference might be based on a biologically determined advantage for processing the left hemispace [42], [77], [78], and an advantage in the processing of increasing order [42], [77], [78].

While reading/writing direction has received most of the attention in order to account for the specific directionality in the number-space mapping because it determines specific directional biases in the exploration of space [42], [77], [78], culture might not be the only determinant of the spatialization of numbers. In fact, non-human species have been shown to display a specific spatial bias when performing ordinal tasks involving numerical information [48], and specifically when spatial and numerical information are congruently combined [49]. The left bias shown by chicks is thought to reflect right hemispheric dominance in visuo-spatial processing, resulting in the left hemifield guiding the birds' behavior (see [50] for similar results in fish). A similar relation between hemispheric lateralization and attentional biases regulating the asymmetrical exploration of space is present in humans. Indeed, human adults manifest the pseudoneglect phenomenon, which consists of a small but consistent leftward bias in visuo-spatial tasks such as the line bisection task [79]. This phenomenon is interpreted in terms of right hemispheric dominance, whereby the right hemisphere would be most activated when performing a visuo-spatial task, with a consequent processing bias towards the contralateral hemifield [80]. Although cultural factors can indeed modulate this leftward attentional bias [81], the baseline starting point is thought to be a right hemisphere specialization for visuo-spatial processing [82], [83], [84].

So far, evidence for the early onset of hemispheric asymmetries engendering leftward biases towards an asymmetrical exploration of space in infancy is limited, although available literature provides some hints for this phenomenon (e.g., [52]; see [51] for a review). Classical behavioral studies on infants' visual exploration indicate that at birth horizontal scans are wider and more frequent than vertical scans [53], suggesting that visual exploration and stimulus detection are easier along the horizontal than the vertical orientation. Moreover, some evidence suggests that a timing asymmetry may exist in the maturation of cerebral hemispheres, with a temporal advantage for the right over the left hemisphere [51], which may pull the attentional system toward left peripersonal space. Electrophysiological and behavioral studies on infants' face perception abilities show an early right hemisphere advantage in the electrocortical responses evoked by faces [85], [86], and a leftward bias in visual exploration of these stimuli (e.g., [87], [88]), and two recent studies have suggested that, from early in development, processing of non-symbolic numerical quantities is lateralized to the right hemisphere [89], [90]. Although limited, this evidence can offer insights into the spatial exploration of space in early stages of development.

Together with a biologically determined advantage for processing the left hemispace [42], [77], [78], the early advantage in the processing of increasing order (see [91] for discussion), recently reported in 4-month-old infants [64], [73], might account for the finding that it is increasing, and not decreasing order, what is associated to the left-to-right spatial displacement. Together, early biases towards an asymmetrical exploration of space brought about by hemispheric asymmetries and towards an asymmetrical processing of ordinal information might constitute the building blocks of a mental mapping where numbers are associated to different spatial positions, with exposure to cultural conventions modulating both biases across the lifespan.

By showing that the association between numerical order and oriented space takes place before exposure to formal symbolic or mathematical instruction has occurred and when the modulating effects of culturally-shaped routines, such as counting or even ‘reading’ illustrated books [92], are minimized, the current results add to existing findings in non-human animals to suggest that the association between numerical order and oriented space is not merely a product of human invention. The present findings suggest that this association stems from early biases present from infancy in both the specific and combined processing of numerical, ordinal and spatial information, with a possible influence of cultural factors engendered by 7 months of interactions with adult caregivers who are likely to structure the environment for their children in many different ways, thus influencing the direction in which infants explore the external space.
